# Supplementation of Labneh with Passion Fruit Peel Enhanced Survival of *E. coli* Nissle 1917 during Simulated Gastrointestinal Digestion and Adhesion to Caco-2 Cells

**DOI:** 10.3390/foods11111663

**Published:** 2022-06-06

**Authors:** Mohamed Samir Darwish, Noha A. Abou-Zeid, Ebtihal Khojah, Huda A. AL Jumayi, Garsa A. Alshehry, Eman H. Algarni, Asmaa A. Elawady

**Affiliations:** 1 Dairy Microbiology Laboratory, Dairy Department, Faculty of Agriculture, Mansoura University, Mansoura 35516, Egypt; asmaa-elawady@mans.edu.eg; 2 Veterinary Medicine Directorate, Mansoura 35516, Egypt; nanoodr@gmail.com; 3 Department of Food Science and Nutrition, College of Science, Taif University, P.O. Box 11099, Taif 21944, Saudi Arabia; eykhojah@tu.edu.sa (E.K.); huda.a@tu.edu.sa (H.A.A.J.); garsa.a@tu.edu.sa (G.A.A.); eman1400@tu.edu.sa (E.H.A.)

**Keywords:** functional food, labneh, probiotic, passion fruit peel, passion fruit, *Escherichia coli* Nissle 1917, fermented milk

## Abstract

Passion fruit peel powder (PFPP) was used to supplement the probiotic labneh to increase the activity of *Escherichia coli* Nissle 1917 (EcN) during production and storage. Labneh was manufactured with PFPP (0.5% and 1%) and analyzed at 0, 7, and 15 days of cold storage for postacidification and sensory properties and viability of EcN, survival of EcN to simulated gastrointestinal tract stress, and adhesion potential of EcN to Caco-2 cells. Acidification kinetics during fermentation showed that supplementation with PFPP reduced the time needed to decrease pH and reach the maximum acidification rate. PFPP addition contributed to postacidification of labneh during storage. PFPP had a beneficial effect (*p* < 0.05) on counts of EcN in labneh during different storage periods. Consumer preference expectations for labneh enriched with PFPP (0.5% and 1%) were higher than those for the control. PFPP provided a significant protective action for EcN during simulated gastrointestinal transit and had a positive effect on EcN adhesion to Caco-2 cells in vitro, although this decreased during storage with labneh. Labneh supplementation with PFPP can be recommended because of the positive effect on EcN viability and the high nutritional value, which may increase the appeal of the product to consumers.

## 1. Introduction

Consumers have become increasingly aware of the positive influences of food on health promotion, and this has led to a development in the global market of functional foods. These are foods that can be considered dietary items, which, besides providing energy and nutrients, beneficially modify one or more targeted functions in the body by decreasing the risk of disease and/or by improving a specific physiological response [1]. Foods that are fermented by probiotic bacteria are considered as being functional foods [1].

Probiotic bacteria are live microorganisms that provide positive impacts to the host when ingested in appropriate numbers. Studies show that probiotic bacteria exert beneficial influences on the gut and the immune system, reduce symptoms associated with irritable bowel syndrome, decrease adverse effects of antibiotics, help alleviate abdominal pain from lactose intolerance, and have antitumor and antimicrobial characteristics [2].

*Escherichia coli* Nissle 1917 (EcN) is a Gram-negative bacteria with probiotic characteristics that was isolated by Alfred Nissle from the feces of soldiers who had not been infected during a Shigellosis outbreak [3]. EcN lacks genomic genes for virulence factors when compared with those in pathogenic *E. coli* [4]. Additionally, EcN has been used to colonize the intestinal tract and modify the microbiota balance and intestinal homeostasis [5,6]. EcN has been improved as a probiotic product under the trademark name Mutaflor, and this has been widely used in Europe to treat infectious diarrhea and intestinal inflammation [7]. Several studies have proven the immunomodulatory mechanisms of EcN, including an increase in the secretion of mucin and immunoglobulin A, induction of antibacterial peptide expression, enhancement of the anti-inflammatory immune response, and promotion of the intestinal barrier [8]. Several studies have indicated the possibility of using EcN in the production of functional fermented milk [9,10].

Dairy products such as cheese, fermented milk, labneh, and yogurt are the most important carriers of probiotic bacteria in the probiotic food industry [11]. Strained/concentrated yogurt is known as labneh in the Middle East, especially in Egypt, where it represents an essential part of the family diet [12]. Labneh is manufactured by removing a proportion of the whey from yogurt until the total solid and fat contents are increased from 23% to 25% and 9% to 11%, respectively [12]. Labneh is a creamy white paste that has a soft and spreadable texture, with a taste between that of cottage cheese and sour cream. Furthermore, labneh has a strong flavor that is mainly modified by diacetyl generated over fermentation [12].

Fermented milk products can be supplemented with many ingredients to improve their nutritional benefits and stimulate the growth and activity of probiotic bacteria during refrigerated storage periods [13]. These bioactive components can maintain probiotic bacterial numbers higher than the minimum recommended level and meet consumer needs for new functional dairy products [13]. Additionally, these components may also increase the passage of probiotics through the gastrointestinal (GI) tract by increasing their resistance to enteric juices and gastric acid [14].

Tropical fruits are a plentiful source of minerals, antioxidants, and vitamins. The passion fruit (*Passiflora edulis* Sims f. *flavicarpa* Deg.) is a tropical fruit that is highly appreciated worldwide [15]. Passion fruit peel has been attributed to have functional characteristics with hypocholesterolemic, antihypertensive, and antidiabetic activities [16] and is known to have a high carbohydrate and pectin content (56% and 10%–20% dry matter, respectively); the pectin component is an important soluble fiber that is recognized as having a prebiotic activity. Passion fruit peel also has a relatively high ash content (approximately 8.7% of dry matter) [16]. The pericarps from passion fruit are rich in functional active substances, such as phenols, flavonoids, and dietary fiber, and have been applied in the improvement of functional food components to become an important investigation trend in a circular economy [17]. Several studies have used passion fruit pericarps to decrease the incubation time of probiotic yogurt [16]. Additionally, polyphenols are known for their prebiotic action on EcN [18]. However, few studies have addressed supplementing fermented milk with passion fruit pericarp, and there is a lack of information about the protective effect of passion fruit peel on probiotic bacteria during GI digestion [16]. This study evaluated the possibility of manufacturing probiotic labneh using passion fruit peel powder (PFPP) and focused on the influence of the addition of PFPP on the fermentation kinetics and post-acidification of probiotic labneh as well as the sensory properties of labneh and EcN counts during cold storage. Furthermore, the survival of EcN to simulated GI digestion and the adhesion of EcN to intestinal cells was also assessed.

## 2. Materials and Methods

### 2.1. Materials

#### 2.1.1. Chemicals and Culture Media

Pepsin, lipase, bovine bile, pancreatin, Dulbecco’s modified Eagle’s minimum (DMEM), nonessential amino acid solution, streptomycin, penicillin, Triton X-100, and fetal bovine serum were purchased from Sigma Aldrich (St. Louis, MO, USA). MacConkey agar, tryptone soya broth (TSB), tryptone soya agar (TSA) and tissue culture media were purchased from Thermo Fisher scientific (Cairo, Egypt).

#### 2.1.2. Bacterial Strain and Cell Line

*Escherichia coli* strain Nissle 1917 (EcN) was taken from stock strains collection of dairy microbiology laboratory (Dairy department, Faculty of Agriculture, Mansoura University, Egypt). EcN were cultured in TSB at 37 °C for 20 h. Individual colonies were then transferred to MacConkey agar and then incubated at 37 °C for 20 h. After microscopic control, EcN was three times re-inoculated and finally grown on TSA. Working culture of EcN was propagated 3 times using a 2% (vol/vol) inoculum in TSB at 37 °C for 20 h before use. Caco-2 cell line (ATCC HTB-39) was obtained from American Type Tissue Culture Collection (ATCC).

### 2.2. Preparation of PFPP

Passion fruit peels were dried in an air drier at 55 °C until a constant weight was reached. The dried peels were then ground into a fine powder using a domestic grinder (BRAUN MQ 60), followed by sieving through a mesh sieve to obtain a particle size of <42 µm to ensure that PFPP could be easily reconstituted in milk.

### 2.3. Production of Probiotic Labneh

Probiotic labneh was produced according to Balabanova et al. [19] with slight modification. Briefly, whole milk powder was reconstituted in distilled water to 12 g/100 g of total solids. The mixture was homogenized with a blender (Braun MQ785 Multi Quick 7 Hand Blender) for 2 min, followed by heating at 95 °C for 15 min and cooling to 40 °C; this was followed by adding different concentrations of PFPP (0.5% and 1%) to the milk, which was then inoculated with *E. coli* Nissle 1917 (2% *v*/*v*). The inoculated mixture was incubated at 37 °C until complete coagulation. The resulting curd was refrigerated and filtered through cloth bags at 5 °C for 12 h to concentrate the fermented curd by removing the whey from the curd; NaCl was then added to 1%. The obtained labneh was transferred into small plastic jars (200 mL) and stored at 5 °C for 15 days. Sample analysis was conducted in triplicates at 0, 7, and 15 days during the refrigerated storage.

### 2.4. Kinetics Parameters of Probiotic Labneh

The fermentation kinetics were determined according to Casarotti, Carneiro and Penna [13]. The rate of change for pH values during probiotic labneh fermentation was assessed every 30 min until the final pH reached 4.5. Five parameters were then evaluated: (1) the maximum rate of acidification (*Vmax*), expressed in 10^−3^ pH min^−1^; (2) the time at which *Vmax* was reached (h); (3) the time (h) required to reach pH 5.0 (T_pH 5.0_); (4) the time (h) required to reach the end of fermentation (pH 4.5) (T_pH 4.6_); and (5) pH at *Vmax* (pH *_Vmax_*).

### 2.5. Postacidification of Probiotic Labneh

The postacidification of labneh was determined at 1, 7, and 15 days of refrigerated storage by measuring the sample pH in triplicate. Additionally, the acidity in tested samples was assessed using titration with 0.1 N NaOH; the total acidity was expressed as lactic acid percent/100 g [13].

### 2.6. Microbiological Analyses of Probiotic Labneh

Cell counts of *E. coli* Nissle 1917 for each treatment were determined in triplicate at 1, 7, and 15 days of cold storage by using the pour plate technique. Briefly, 1 g of labneh sample was diluted in 9 mL sterile saline (0.85% NaCl). Bacterial counts were enumerated using MacConkey agar after incubation for 24 h at 37 °C. This result was expressed as colony-forming units/g of labneh (Log CFU g^−^^1^).

### 2.7. Sensory Analyses of Probiotic Labneh

Sensory analysis of probiotic labneh samples was evaluated at 23 °C under white light and in individual booths. The evaluation of sensory properties of tested samples was conducted in terms of appearance, texture, color, flavor, sourness, and general acceptability according to a 9-point hedonic scale, where 9 = like extremely, 8 = like very much, 7 = like, 6 = like slightly, 5 = neither like nor dislike, 4 = dislike slightly, 3 = dislike, 2 = dislike very much, and 1 = dislike. Consumer acceptance of tested samples was scored by 25 faculty members and students from the Faculty of Agriculture, Mansoura University, Mansoura, Egypt [20].

### 2.8. In Vitro Evaluation of EcN Survival under Conditions Simulating the GI Tract

The effect of PFPP on EcN survival in labneh in GI tract-simulated conditions was assessed as reported by Buriti et al. [21], with modification. Briefly, 25 g of labneh was suspended in 225 mL of 0.85% NaCl, and then 10 mL of this mixture was transferred to a sterile flask. The pH was adjusted to between 1.9 and 2.1 using 0.5 M HCl, followed by the addition of lipase (0.9 mg/L) and pepsin (3 g/L) to the mixture. Samples were incubated for 2 h at 37 °C (gastric phase). In the next stage, the pH of the samples was adjusted to between 4.4 and 5.3 using an alkaline solution (14 g of PO_4_H_2_Na.2H_2_O and 150 mL of 1 M NaOH dissolved in distilled water and diluted to 1 L). Pancreatin (from the porcine pancreas) and bovine bile were added to 1 and 10 g/L, respectively. Samples were incubated for 2 h at 37 °C (enteric phase 1). Finally, the sample pH was readjusted to between 7.0 and 7.3 using the same alkaline solution. The pancreatin and bile concentrations were adjusted to 1 and 10 g/L, respectively, and samples were reincubated for 2 h at 37 °C (enteric phase 2). Aliquots of 1 mL were collected from triplicate samples at the beginning of the assay and after the end of each stress stage and then precipitated by centrifugation (6000× *g* for 15 min) and washed three times by resuspension in 3 mL of phosphate-buffered saline (PBS) (pH 7.5), followed by reprecipitation as described above. EcN cells were then resuspended in PBS (3 mL), and bacterial survival was determined as described in the microbiological analysis section.

### 2.9. Adhesion of EcN to Caco-2 Cells

The Caco-2 cell line (ATCC HTB-39) was cultured in DMEM enriched with a 1% solution of nonessential amino acids, a mixture of streptomycin (100 μg/mL) and penicillin (100 IU/mL), and 20% fetal bovine serum. An assessment of cell adhesion was conducted as reported by Ranadheera et al. (2012), with slight modifications. Briefly, Caco-2 cells (10^5^ cells/well) were seeded into 24-well tissue culture plates and incubated in 5% CO_2_ at 37 °C until a confluent monolayer had formed; media was changed with antibiotic-free medium (DMEM) 24 h before adhesion was assessed. All traces of the medium were then removed from the monolayer by washing once with PBS (pH 7.4). An aliquot of 1 mL of each labneh sample was added to confluent Caco-2 cell monolayers, followed by incubation in 5% CO_2_ at 37 °C for 2 h. Cells were then washed at least three times with PBS to eliminate nonadherent bacterial cells. Triton X-100 (1 mL) was added to each well to detach bacterial cells. To evaluate adhesion potential, the suspension (1 mL) from each well was then transferred to a sterilized tube containing 9 mL of sterile saline (0.85% NaCl), serially diluted, and plated on the MacConkey agar in triplicate. The percentage of adhered bacteria was calculated by dividing the viable bacterial counts adhered to the cell layers by the viable bacterial counts from the labneh before the adhesion assay. The experiment was conducted in triplicate.

### 2.10. Statistical Analysis

All tests were carried out in triplicate. ANOVA test with the significance level at *p* < 0.05 was used to evaluate the changes in kinetics parameters, post-acidification, numbers of EcN, survival of EcN under conditions simulating the GIT, and adhesion of EcN to Ca-co-2 cells. The data were shown as average ± standard deviation. The Duncan’s multiple range tests was used for determination of significant differences between values. SPSS Statistics software V.23 was used to evaluate all statistical tests in the current study. A principal components analysis (PCA), hierarchical cluster analysis (HCA) and contour plot of preference and preference map were performed using the XLSTAT program.

## 3. Results

### 3.1. The Chemical Composition of PFPP

The chemical composition of PFPP is presented in Table S1. The obtained results showed that PFPP is a rich source of carbohydrate, fiber, ash, protein and lipid. PFPP showed a high content of carbohydrates compared with other compounds, probably due to the high content of pectin (31.18 ± 0.94).

### 3.2. The Influence of PFPP Addition on the Fermentation Kinetics of Labneh

**Figure 1:**
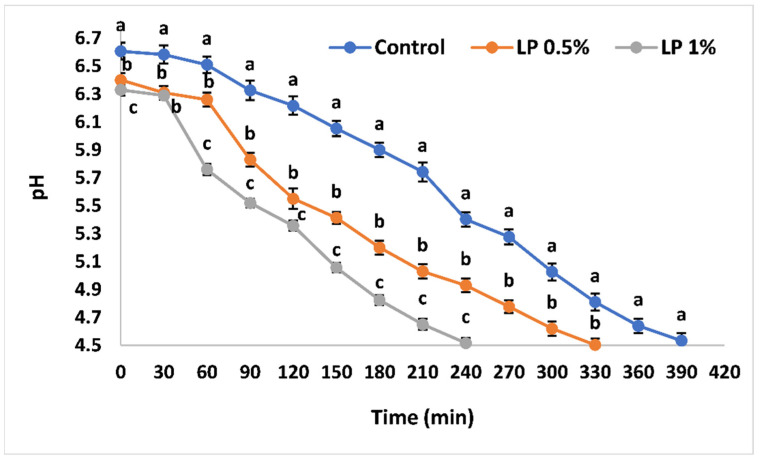
Effect of PFPP on acidification kinetic profiles of labneh. Different lowercase superscripts indicate significant (*p* < 0.05) differences between the values for different treatments. LP: Labneh supplemented with PFPP. Vertical bars present the standard division of the treatment means.

Changes in pH of inoculated milk were determined during the fermentation process (Figure 1). The addition of PFPP significantly decreased (*p* ˂ 0.05) the initial pH of milk with 0.5% or 1% PFPP from 6.61 ± 0.03 to 6.4 ± 0.02 and 6.33 ± 0.02, respectively. Generally, the labneh without or with PFPP showed similar trends in pH changes. The pH of labneh enriched with 0.5% PFPP or control underwent a minor decrease in the first 60 min, but then decreased rapidly until the end of fermentation. However, the pH of labneh enriched with 1% PFPP decreased within the first 30 min, which was followed by a sharp decrease until the end of fermentation. The effect of PFPP addition on the tendency of changes in the labneh pH during the fermentation processes was consistent with the labneh fermentation kinetic trends (Table 1). There was a decreasing trend in time required to reach pH 5.5, 5.0, and 4.5 (Figure 1) with an increasing trend in the maximum acidification rate with the increase in PFPP (Table 1). The maximum acidification rate during fermentation for control labneh and labneh enriched with 0.5% and 1.0% PFPP were 11.33 × 10^−3^ ± 0.67, 14.2 × 10^−3^ ± 1.3, and 17.67 × 10^−3^ ± 0.88 pH/min, respectively.

### 3.3. Postacidification and Titratable Acidity

Table 2 shows changes in titratable acidity and postacidification (pH) with storage of labneh. At zero time, the labneh pH ranged from 4.37 to 4.48. Significant (*p* < 0.05) differences were present between the pH of labneh enriched with 0.5% of PFPP (4.42 PFPP labneh and 4.48 control) and labneh enriched with 1% of PFPP (4.37 PFPP labneh and 4.48 control) (*p* < 0.05). Titratable acidity varied from 0.86 to 1.05 mg lactic acid/g. There was also a significant increase in acidity induced by PFPP addition to labneh (*p* < 0.05).

After 7 days of storage, all the labneh samples showed a significant reduction in pH (*p* < 0.05), which ranged from 4.32 to 4.39 among the treatments, which continued for the full 15 days of treatment (*p* < 0.05) (Table 2). The decline of labneh pH was directly proportional to the addition of PFPP (Table 2). After 7 days of storage, all samples exhibited a marked increase in their titratable acidity, although the labneh samples supplemented with PFPP still had a higher level of acidity compared with that of the control (*p* < 0.05). At both 7 and 15 days, the labneh enriched with 1% of PFPP had the highest values of average titratable acidity (*p* < 0.05).

### 3.4. Viability of EcN during Storage of Labneh

**Figure 2:**
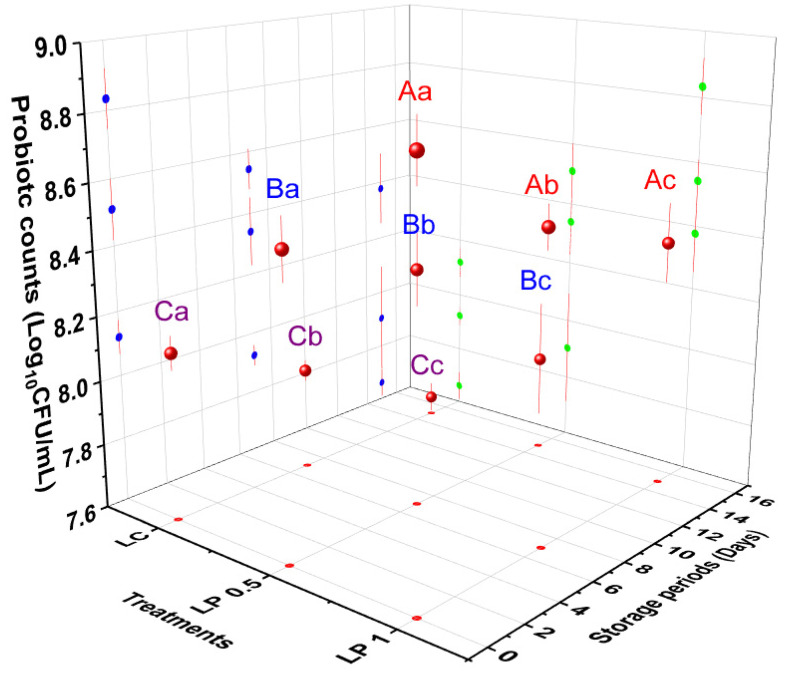
Counts of EcN in labneh during refrigerated storage. LC (labneh without PFPP), LP 0.5 (labneh with 0.5% of PFPP and LP 1 (labneh with 1% of PFPP). Different uppercase superscripts indicate significant (*p* ˂ 0.05) differences between the values for various concentrations of PFPP incorporation. Different lowercase superscripts indicate significant (*p* ˂ 0.05) differences between the values during the shelf-life of labneh. Vertical bars show the standard division of the treatment means.

During the storage period, the population of EcN ranged from 7.66 to 8.12 log_10_ cfu/mL in labneh without PFPP (Figure 2) and showed a decline of 0.46 log_10_ cfu/mL by the end of storage. The EcN population in labneh enriched with 0.5% PFPP ranged from 7.9 to 8.51 log_10_ cfu/mL during the 15-day storage period (Figure 2), reducing to approximately 0.61 log_10_ cfu/mL, whereas the population in labneh enriched with 1% PFPP ranged from 8.38 to 8.83 log_10_ cfu/mL. Regarding the control, PFPP had a positive influence (*p* < 0.05) on the EcN population in labneh during different times of the storage period, where the counts of EcN increased significantly (*p* ˂ 0.05) from 7.66 to 8.33 log_10_ cfu/mL in PFPP incorporation at day 0 (Figure 2).

### 3.5. Cluster Analysis and Preference Map of Probiotic Labneh Enriched with PFPP

**Figure 3:**
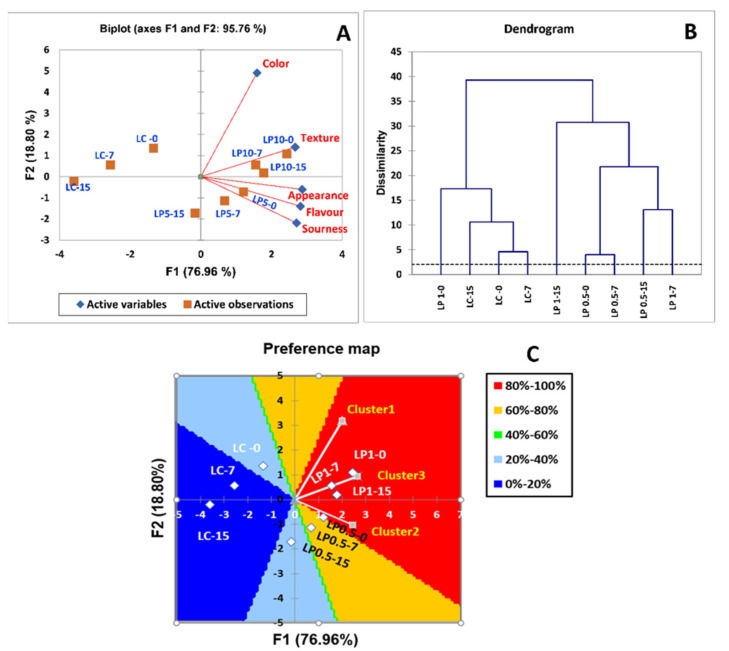
(**A**) Representation of attributes and samples in the first two dimensions of the correspondence analysis (CA) of labneh enriched with PFPP. (**B**) Dendrogram derived from the hierarchical cluster analysis (HCA) on the representation of labneh enriched with PFPP. (**C**) Contour plot of preference and preference map of consumers clusters for different treatments: LC-0 (Labneh without PFPP at day 0), LC-7 (Labneh without PFPP after 7 days of storage), LC-15 (Labneh without PFPP after 15 days of storage), LP 0.5-0 (Labneh with 0.5% of PFPP at day 0), LP 0.5-7 (Labneh with 0.5% of PFPP peel after 7 days of storage), LP 0.5-15 (Labneh with 0.5% of PFPP after 15 days of storage), LP 1-0 (Labneh with 1% of PFPP at day 0), and LP 1-7 and LP 1-15 (Labneh with 1% of PFPP after 7 and 15 days of storage, respectively).

Principal component analysis of appearance, color, texture, flavor, and sourness properties of labneh enriched with different concentrations of PFPP (0.5% and 1%) at different times of storage period (zero time, 7, and 15 days) explained 95.76% of the variability in two PCs (Figure 3A). PC1 (76.96%) included appearance, texture, flavor, and sourness. However, the second dimension (18.80%) was mainly associated with color (Figure 3A). The treatments were distributed into three categories. The first (LP 1-0, LP 1-7, and LP 1-15) and second (LP 0.5-0, LP 0.5-7, and LP 0.5-15) categories were located on the positive value side of PC1, while the third category was positioned on the left side of PC1 (LC-0, LC-7, and LC-15). The first category was characterized by high scores for appearance, texture, flavor, color, and sourness, followed by the second category (Figure 3A). The third category was characterized by the lowest values of all parameters (Figure 3A).

To verify whether the participants enrolled in the study formed preference clusters, hierarchical cluster analysis was used. This has the potential to imagine the existence of two parts, in both regions, as shown in the obtained dendrogram (Figure 3B). One group was formed by control labneh samples at different storage periods and labneh enriched with 1% of PFPP at day 0 (LP 1-0, LC-0, LC-7, and LC-15), and another group was formed by labneh samples enriched with different concentrations of PFPP at different storage periods (LP 1-15, LP 0.5-0, LP 0.5-7, LP 0.5-15, and LP 1.7). These results confirm that consumers separated the samples (except LP 1-0) by the PFPP used. This suggests that consumers noticed differences between labneh samples without PFPP (control) from samples enriched with PFPP.

Consumer division according to acceptance data provided three evaluation clusters: cluster 1 (40% of consumers), cluster 2 (30% of consumers), and cluster 3 (30% of consumers). Figure 3C presents the results of the preference mapping performed on the general acceptance data. Contour graphs show the preferential zones in relation to the treatments, where the dark blue zone (0% to 20%) and the light blue zone (20% to 40%) are the lowest preferred regions, followed by the green zone (40% to 60%); the yellow and red zones (60% to 80% and 80% to 100%, respectively) are those with the highest preferences. Three consumer clusters were determined according to the preference for the labneh samples. Vector models were used for all three clusters with no ideal points or saddle. Consumers in three clusters displayed a high preference for labneh enriched with 1% of PFPP at 0, 7, and 15 days of storage (LP 1-0, LP 1-7, and LP 1-15), reaching 80% to 100% of consumers (Figure 3C). However, their preference for labneh without PFPP (control) at different times of storage (LC-0, LC-7, and LC-15) was low (Figure 3C). Generally, consumer preference expectations for labneh enriched with different concentrations of PFPP (0.5% and 1%) were higher than those for the control.

### 3.6. Survival of EcN against the Simulated GI Stress

**Figure 4:**
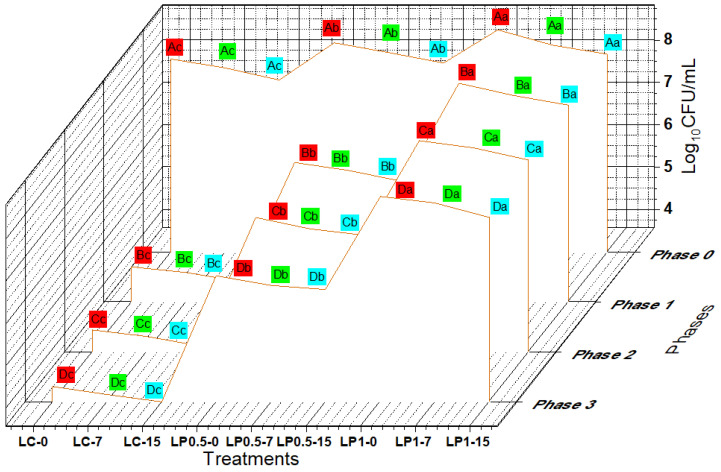
Survival of EcN (viable count; log_10_ cfu/mL) in probiotic labneh during cold storage before (phase 0) and during exposure to in vitro simulated gastric conditions (phase 1), and enteric conditions (phase 2, pH 4.3–5.2) and simulated enteric conditions (phase 3, pH 7.0–7.3). LC-0 (Labneh without PFPP at day 0), LC-7 (Labneh without PFPP after 7 days of storage), LC-15 (Labneh without PFPP after 15 days of storage), LP 0.5-0 (Labneh with 0.5% of PFPP at day 0), LP 0.5-7 (Labneh with 0.5% of PFPP peel after 7 days of storage), LP 0.5-15 (Labneh with 0.5% of PFPP after 15 days of storage), LP 1-0 (Labneh with 1% of PFPP at day 0), and LP 1-7 and LP 1-15 (Labneh with 1% of PFPP after 7 and 15 days of storage, respectively). Different lowercase superscripts indicate significant (*p* ˂ 0.05) differences between the values for various concentrations of PFPP incorporation. Different uppercase superscripts indicate significant (*p* ˂ 0.05) differences between the values during passage in the simulated GI-tract condition.

We then assessed the tolerance of EcN in probiotic labneh that had been exposed to simulated GI conditions (Figure 4). This probiotic strain was incubated in stress conditions that simulated the majority of effects that impact the tolerance of the ingested probiotic strains during their travel through the digestive system (Figure 4). To mimic the sequential gastric movement of microorganisms through the intestine during digestion, we assessed three pertinent conditions: the effect of pepsin, acidic conditions (phase 1), and addition of pancreatin and bile salts (phases 2 and 3). EcN had a low gastric tolerance in the labneh control, whereas a beneficial effect was provided by the enrichment of labneh with different concentrations of PFPP as a carrier dairy matrix, and this improved the retention of EcN viability during gastric transit with simulated gastric juice at pH 2.0. The viability of EcN in gastric juice at pH 2.0 increased with the increasing concentration of PFPP (Figure 4). However, EcN viability decreased in all treatments with the extended storage of functional labneh. Overall, EcN in plain labneh after 15 days of cold storage period exhibited the lowest tolerance to gastric juice (Figure 4). In relation to the second and third phases for simulated intestinal juice, the inclusion of pancreatin and bile salt had a notable effect on decreasing EcN viability during in vitro transit of the small intestine (Figure 4). This influence was significantly evident after exposure to simulated intestinal juice (phases 2 and 3) on EcN viability at the end of storage periods regardless of the concentration of PFPP (Figure 4). With a decline in the EcN viable counts limited to ≤ 3.6 log in labneh enriched with PFPP, across the final phase of exposure to in vitro simulated GI conditions. Overall, we detected a significant decrease (*p* ≤ 0.05) in the population of EcN during transit of the food matrix through simulated GI-tract stress (Figure 4). The protective influence of PFPP at different concentrations on the survivability of EcN was significant from a bacteriological perspective, with a variation of 1 log_10_ CFU/mL among the populations of EcN in supplemented labneh and control at the completion of the in vitro experiment. The reduction in the EcN population after the in vitro assay was affected by continued storage (Figure 4).

### 3.7. EcN Adhesion to Intestinal Cell In Vitro

**Figure 5:**
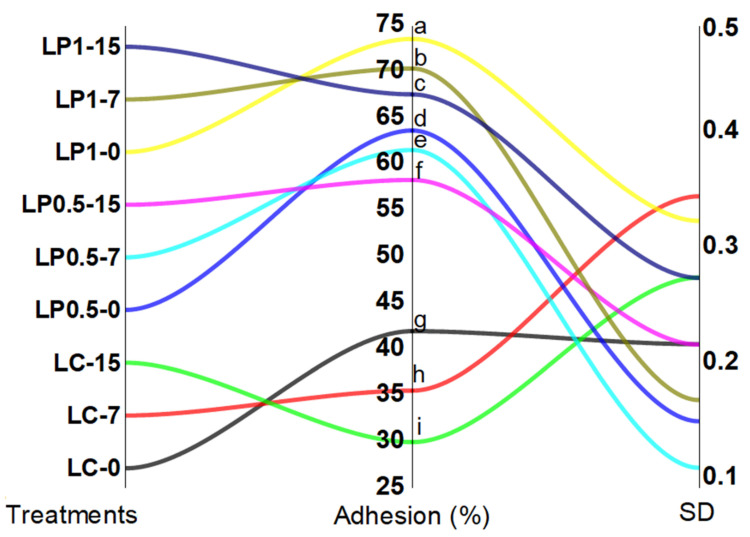
The effect of fortification of labneh with PFPP on the percentage of EcN adhesion to Caco-2 cells at different times of cold storage. LC, LP 0.5, and LP 1 denote plain labneh, labneh enriched with 0.5% of PFPP, and labneh enriched with 1.0%, respectively. The numbers 1, 7, and 15 indicate the time (days) of the cold storage period. ^a–i^ Different lowercase letters in the figure for each treatment indicate significant differences between treatments (*p* ˂ 0.05).

Finally, we assessed the adhesion of EcN to intestinal cells in vitro (Figure 5). EcN adhesion ranged from 30.1% to 73.63% with labneh enriched with 1% PFPP and was highest at day 0 (73.63%), whereas EcN in plain labneh after 15 days of refrigerated storage period had the lowest adhesion potential (30.1%). There was a significant difference (*p* ˂ 0.05) in EcN adhesion between the labneh treatments at different storage times (Figure 5). The adhesion potential of EcN significantly (*p* ˂ 0.05) increased with increasing PFPP content (Figure 5).

### 3.8. Effect of Digestion on the Total Phenolic Content of PFPP

The total phenolic content of PFPP were shown in Figure S1. The highest total phenolic content (TPC) of PFPP was found in labneh enriched with 1% of PFPP after 15 days of storage period, followed by labneh enriched with 1% of PFPP after 7 and 0 days of storage periods. The plain labneh (without PFPP) displayed a slight increase in TPC until the end of storage (Figure S1).

After in vitro simulated GI digestion. TPC of labneh enriched with PFPP were significantly increased (Figure S1). Labneh enriched with 1% of PFPP at zero-time showed the highest TPC compared with other treatments. The TPC after in vitro simulated digestion was inversely proportional to storage progressed.

## 4. Discussion

Different types of fermented dairy products can act as carriers for delivering probiotic strains through the food matrix. *Bifidobacterium animalis* subsp. *lactis*, *Lactobacillus acidophilus*, and *Lactobacillus casei* strains predominate in commercial probiotic products [22,23]. In this study, we analyzed the unusual probiotic strain (*E. coli* Nissle 1917) that is used for the production of probiotic labneh enriched with PFPP for tolerance to GI-tract stress and the ability to interact with human intestinal cells.

The results obtained from chemical composition of PFPP in this study were similar with dos Reis et al. [24], who reported that the peels of purple, orange and yellow passion fruits were shown to be richer in pectin, total dietary fiber, proteins and lipids.

The maximum acidification rate during fermentation was positively correlated with the concentration addition of PFPP (*p* < 0.05). This behavior may be due to the high content of organic acids and polyphenols in passion fruit [25]. This study shows that supplementation of labneh with PFPP could increase the fermentation rate and thereby reduce fermentation and solidification time. According to Varghese K and Mishra [26], the total solids content of the fermented milk is directly proportional to fermentation time because of the high buffering capacity present. This observation, which is undoubtedly valid for increasing total solid with milk derivatives, does not appear to be appropriate to increase in total solids induced by the addition of PFPP. However, the individual capacity of different probiotic strains to assimilate nutritional ingredients of milk can lead to different acidification profiles [27]. McCann et al. [28] also reported that the addition of carrot was responsible for an approximate 1 h decline in the fermentation time of yogurt fermented by *Streptococcus thermophilus* and *Lactobacillus delbrueckii* ssp. *bulgaricus*.

In this study, we detected significant differences (*p* ≤ 0.05) in titratable acidity increase and pH reduction during the shelf-life for all tested labneh samples. The fermentation of residual lactose and lactic acid production occur during the cooled storage period of labneh as a result of the metabolic activity of the starter culture [29]. Consistent with our findings, fermented milk enriched with 1%, 2%, and 3% of pea flour resulted in a higher decline in pH during storage compared with the control product [30]. However, other findings found that the addition of the fiber (wheat, apple, inulin, bamboo, orange inulin, date, wheat bran, or lemon) did not affect the acidity and pH of yogurt samples [31,32,33]. Thus, postacidification of fermented milk appears to rely on the type of starter culture and the components in the milk base [13].

Despite the variations detected in the EcN population, the minimum beneficial dose of probiotic bacteria between 10^6^ and 10^9^ cfu/mL in a functional dairy product [34] was achieved in all treatments until the end of the labneh storage. In agreement with this finding, Kailasapathy et al. [35] found that there was a reduction in the population of the probiotic strain in fermented milk enriched with passion fruit juice. However, Darwish et al. [9] found that the population of EcN increased until the end of the cold storage period for yogurt enriched with Cape Gooseberry. Furthermore, do Espirito et al. [16] also reported that the numbers of *L.* *acidophilus* L10 were not affected by PFPP addition to yogurt. The improvement of EcN survival during the storage period of labneh enriched with PFPP has been attributed to high contents of pectin and phenolic compounds and fatty acids [16].

The present study shows that the addition of PFPP improved the water-holding capacity and gel strength of labneh according to sensory evolution of labneh, resulting in a decreased separation of whey from the labneh, which increased the consumer acceptability for labneh. We detected an increasing trend for general acceptability, flavor, and aroma with the addition of PFPP. The results of our study are consistent with those of do Espirito et al. [16], who showed that consistency, firmness, and cohesiveness were increased by the addition of PFPP to yogurt. Ning et al. [20] also reported that the sensory characteristics of yogurt enriched with passion fruit juice were superior to that of control at a concentration of passion fruit juice from 5% to 7.5%.

To evaluate EcN resistance to GI-tract conditions, we have studied the in vitro tolerance under stresses that mimic the normal physiological stress of the GI system, such as lower pH values and the presence of pepsin and of pancreatin and bile salts. This analysis has been previously used to evaluate probiotic strain viability in probiotic preparations and functional dairy products [36,37]. However, to our knowledge, this is the first finding on the general application of this system for the assessment of EcN viability associated with labneh after GI stress. Probiotic strains must reach the intestine at adequate levels to exert their positive health benefits. Therefore, a typical carrier must be appropriate for human consumption, be able to maintain the viability of the probiotic strains during the manufacture and during the shelf-life of the food products, and protect the probiotics during travel through the GI tract to ensure that they are delivered to the colon [38]. Studies have indicated that structures of dietary content and food matrices can interact with different probiotic strains and protect them during passage through the GI system [39,40]. Supplementing labneh with PFPP may have provided suitable protection for the EcN tolerance when exposed to simulated GI conditions by serving as a protective cover against gastric and enteric juices. Thus, the chemical composition of PFPP (lipid, protein, fiber, vitamins, and phenolic compounds) [24] enhanced EcN survival to the mimicked GI stress. This impact was clearly pronounced for the high concentration of PFPP tested against gastric juice. EcN was able to survive in acidic conditions (pH 2.0), whereas growth was not affected by bile salt addition at concentrations between 0.1% and 0.3% [41,42]. In this study, EcN viability remained significantly higher during GI-tract stress when fortified with PFPP compared with that in plain labneh. This was probably due to the protective impact of phenolic compounds of labneh enriched with PFPP in comparison with that of plain labneh [43,44,45,46].

To our knowledge, this is the first study to address the cellular adhesion of EcN suspended in labneh enriched with PFPP to assess the effect of supplementation of PFPP on ingestion of EcN in fermented milk. Using 7–8.8 log_10_ CFU/mL, the level of EcN that adhered to Caco-2 cells was relatively high for all treatments (30 to 73.6%), as reported in other studies using the different species [13,42]. Certain probiotic strains may have greater adhesive ability because of their specific biochemical and physiological biochemical properties [47]. Low adhesion of probiotic strains to intestinal cells leads to an increased excretion rate in feces [48]. Extended storage of probiotic labneh decreased (*p* ≤ 0.05) the capacity of EcN to adhere to Caco-2 cells, and this may be attributed to the low pH in labneh samples at the end of the storage period. The adherence ability of *Enterococcus faecium* strains to IPEC-J2 cells significantly decreased because of their pretreatment at pH 3.0 [49]. Deepika et al. [50] also indicated a decline in the adhesion potential of probiotic strains during storage in food models simulating ice cream and yogurt, and they deduced that the storage period influences the adhesion potential of probiotic strains more than other factors, such as sugar and fat contents. In that study, the extended storage led to a decrease in the hydrophobicity of probiotic strains and was linked to a decline in the adherence potential to Caco-2 cells. Multiple studies have evaluated the adhesion of probiotics to many types of cell lines [51,52]. However, few studies have been conducted to assess the influence of food components on the adherence potential of probiotic strains. Here, we observed that PFPP influenced the adhesion of EcN. The characteristics of cell surfaces of probiotic strains and, consequently, their adhesion to the intestinal cells may be affected by the type of food matrix carrier or specific food components [50,53].

The increase of TPC after exposure to GIT stress has been attributed to some polyphenols with high molecular weight hydrolyzed into other compounds [54]. Additionally, this may be due to generation of new polyphenols substances in oxidized and acidic environment [55]. In addition to, the bound polyphenols substances would release from macromolecular compounds like protein [54]. The EcN resistance to GI-tract condition and adhesion to Caco-2 cells were directly proportional to TPC.

## 5. Conclusions

This study demonstrated that PFPP significantly affected the fermentation kinetics of the probiotic labneh. PFPP addition was conducive to a reduction in pH in the early stage of the fermentation process, which effectively activated the growth of EcN. During the fermentation, PFPP increased the fermentation rate and reduced the fermentation time. The pectin and polyphenols in PFPP exerted a positive effect on the survivability of EcN during in vitro transit through a simulated GI-tract environment. Furthermore, the addition of PFPP improved the survival of EcN during refrigerated storage of labneh. PFPP was also beneficial in increasing the adhesion rate of EcN to intestinal cells and improving the flavor, aroma, and general acceptability of labneh. Hence, it is feasible to produce functional labneh of superior quality by incorporating 0.5% to 1% PFPP. However, more studies are needed to explain the underlying mechanisms whereby PFPP influences EcN survival during transit of the GI tract and the adhesion potential of EcN to Caco-2 cells.

## Figures and Tables

**Table 1:** Effects of PFPP addition at various concentrations on the fermentation kinetics of labneh.

Parameters/Treatments	Control	LP 0.5%	LP 1%
Initial pH	6.61 ± 0.03 ^a^	6.4 ± 0.02 ^b^	6.33 ± 0.02 ^c^
tpH5.5 (min)	228.05 ± 0.23 ^a^	149.3 ± 0.68 ^b^	103.4 ± 1.3 ^c^
tpH5.0 (min)	314.8 ± 0.69 ^a^	230.9 ± 1.32 ^b^	165.4 ± 1.7 ^c^
tpH4.5 (min)	401.5 ± 1.56 ^a^	312.4 ± 2.1 ^b^	227.4 ± 2.1 ^c^
Vmax (10–3pH/min)	11.33 ± 0.67 ^c^	14.2 ± 1.3 ^b^	17.67 ± 0.88 ^a^
Tmax (min)	240 ^a^	90 ^b^	60 ^c^

LP: Labneh supplemented with PFPP. Different lowercase superscripts indicate significant (*p* ˂ 0.05) differences between the values for various concentrations of PFPP incorporation. Results were expressed as the mean of triplicates ± standard deviation (S.D.).

**Table 2:** Titratable acidity and postacidification during the storage period of labneh enriched with PFPP and control.

Treatment	pH			Titratable Acidity (% Lactic Acid)		
	Storage Period (Days)					
	0	7	15	0	7	15
Control	4.48 ± 0.01 ^Aa^	4.39 ± 0.03 ^Ba^	4.32 ± 0.02 ^Ca^	0.86 ± 0.03 ^Cc^	0.99 ± 0.03 ^Bc^	1.08 ± 0.02 ^Ac^
0.5% PFPP	4.42 ± 0.02 ^Ab^	4.35 ± 0.02 ^Bb^	4.25 ± 0.03 ^Cb^	0.95 ± 0.02 ^Cb^	1.08 ± 0.02 ^Bb^	1.16 ± 0.02 ^Ab^
1% PFPP	4.37 ± 0.03 ^Ac^	4.32 ± 0.02 ^Bc^	4.20 ± 0.02 ^Cc^	1.05 ± 0.03 ^Ca^	1.14 ± 0.03 ^Ba^	1.22 ± 0.01 ^Aa^

Results were expressed as the mean of triplicates ± standard deviation (S.D.). Different uppercase superscripts indicate significant (*p* ˂ 0.05) differences between the values during the shelf-life of labneh. Different lowercase superscripts indicate significant (*p* ˂ 0.05) differences between the values for various concentrations of PFPP incorporation.

## Data Availability

All data generated or analyzed during this study are available from the corresponding author on reasonable request.

## Supplementary Materials

The following supporting information can be downloaded at: https://www.mdpi.com/article/10.3390/foods11111663/s1, Figure S1: The total phenolics content of PFPP before and after in vitro gastrointestinal digestion.; Table S1: Proximate composition of PFPP (g/100 g of dry weight—except for moisture) with mean and standard deviation. References [56,57] are cited in the supplementary materials.
