# Supplementary material for: Supplementation of Labneh with Passion Fruit Peel Enhanced Survival of E. coli Nissle 1917 during Simulated Gastrointestinal Digestion and Adhesion to Caco-2 Cells
Source: Foods. 2022 Jun 6;11(11):1663. doi: 10.3390/foods11111663 (PMC9180240; doi:10.3390/foods11111663)
Supplement: Supplementary file 1 [file foods-11-01663-s001.zip › foods-1739161-supplementary.pdf]

2. Materials and Methods

2.1. Chemical Analysis of PFPP

The determination of protein, lipid, ash, total fiber and moisture of PFPP was carried out according to AOAC [56]. Protein content was determined by the Kjeldahl method with a conversion factor of 5.75. Lipids were obtained by cold extraction, and ash was determined in a muffle furnace at 550 °C. Total dietary fiber was determined by the enzymatic–gravimetric method, moisture by gravimetry. Carbohydrate content of PFPP were calculated by the difference of 100 minus the sum of the other proximate components (protein, lipid, ash). The results of all analyses were expressed as %.

2.2. Determination of Pectin Content

The pectin of PFPP was determined using method of Seixas, *et al.* [57] with minor modifications. Each two grams of PFPP was mixed with 50 mL of distilled water. The mixture was acidified by adding the 10% of tartaric acid solution until final pH 2, followed by heating in microwave for 3 min. The filtration of solution by filter paper was carried out and then cooled at 4 °C. Two volumes of absolute ethanol were slowly added to filtrate under magnetic stirring. The mixture was mixed for 15 min and then allowed to rest for 40 min to facilitate the pectin flotation. The pectin was isolated by vacuum filtration. The resulted pectin was transferred to absolute ethanol for overnight. The dehydration of pectin was performed by air-circulated oven at 50 °C until constant weight. The concentration of pectin in PFPP was expressed and gram of pectin per 100 g of PFPP.

2.3. Total Phenolic Content Assay

Aqueous extract of Labneh samples was prepared following a standard procedure [3]. This extract was used to determine the total phenolic content (undigested polyphenols). However, aliquots of 1 mL collected after the enteric phase 2 in human digestion simulation model were used to determine the digested total phenolic content. Spectrophotometric determination of the total phenolic content of the samples was assessed according to the method reported by Darwish et al. [9]. Briefly, a volume of 0.5 mL of each sample was mixed with 0.1 mL Folin–Ciocalteau reagent and 0.5 mL of sodium carbonate 7.5% (w/v). The mixture was incubated for 60 min in the dark at room temperature and the absorbance was recorded at 740 nm, against distilled water as blank. The standard curve was plotted using gallic acid as a reference for the phenolic compound. Total phenolic content in before and after digestion was expressed as μg gallic acid equivalent/mL.

3. Results

Table S1. Proximate composition of PFPP (g/100 g of dry weight—except for moisture) with mean and standard deviation.

	Moisture	Protein	Lipid	Ash	Carbohydrate	Total Fiber	Pecten
**PFPP**	g/100 g of dry weight						
	86.91 ± 0.23	6.8 ± 0.55	5.22 ± 1.12	7.64 ± 0.44	80.34 ± 0.72	16.22 ± 1.2	31.18 ± 0.94

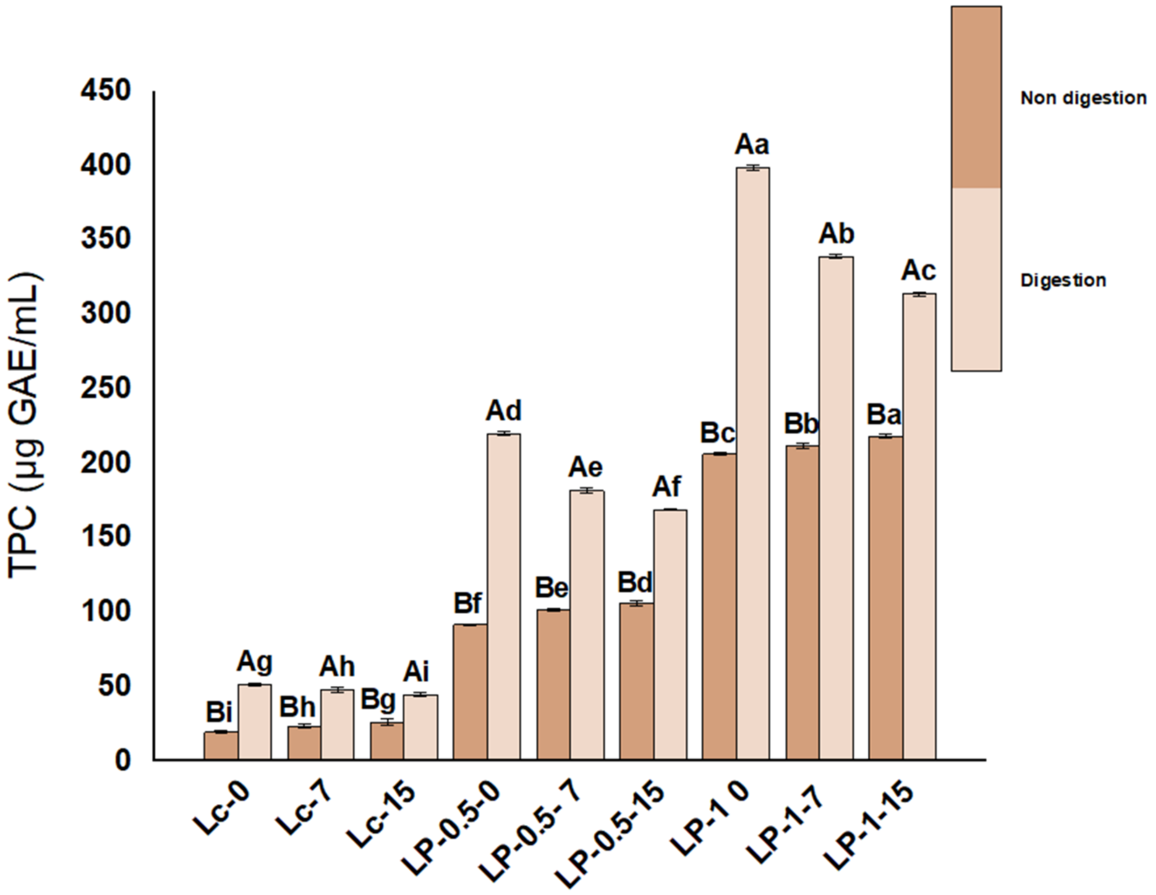

Figure S1. The total phenolics content of PFPP before and after in vitro gastrointestinal digestion. LC, LP5, and LP10 denote plain labneh, labneh enriched with 0.5% of PFPP, and labneh enriched with 1.0%, respectively. The numbers 1, 7, and 15 indicate the time (days) of the cold storage period. Different lowercase superscripts indicate significant ($p < 0.05$) differences between the values for various concentrations of PFPP incorporation. Different uppercase superscripts indicate significant ($p < 0.05$) differences between the values before and after passage in the simulated GI-tract condition.
